# Overcoming cross-incompatibility in genus *Arachis* via *in situ* embryo rescue

**DOI:** 10.1270/jsbbs.24031

**Published:** 2024-12-05

**Authors:** Chun Jiao Jiang, Hao Jie Sun, Jia Kai Li, Wei Jie Qi, Guang Di Yuan, Zhi Wei Wang, Ming Jun Zhang, Xuan Qiang Liang, Chuan Tang Wang

**Affiliations:** 1 Shandong Peanut Research Institute, 126 Wannianquan Rd, Licang District, Qingdao, 266100, China; 2 Shandong Rainbow Agri-Tech Co. Ltd., 8th Floor, Building E, China Food Valley Headquarters, Kaiyuan Street, Hanting District, Weifang, 261100, China; 3 Guangdong Academy of Agricultural Sciences, 18 Jinying West 2nd Street, Tianhe District, Guangzhou, 510641, China

**Keywords:** groundnut, wild species, incompatibility, KASP, transposon element

## Abstract

The cultivated peanut, *Arachis hypogaea* L., is an important source of edible oil and highly digestible protein. Wild incompatible *Arachis* species outside section Arachis are ideal gene reservoirs for genetic improvement of the peanut crop. Among these, *A. glabrata* Benth. stands out for its noted resistance to various stresses. Traditional *in vitro* embryo rescue techniques have been fraught with challenges, including time consumption, resource intensiveness, late intervention timing, and limited effectiveness. In this study, we employed three hormone formulations in an innovative *in situ* embryo rescue approach to facilitate the production of intersectional *Arachis* hybrids. Through this method, hybrid seeds resulting from the crossing of two incompatible species, namely *A. glabrata* and *A. paraguariensis*, with four high-oleic peanut varieties were successfully obtained. Molecular marker analysis and observation of plant characteristics confirmed the hybrid nature of these seeds. This breakthrough represents a significant advancement in expediting the utilization of incompatible wild *Arachis* species in peanut breeding programs. Moreover, the *in situ* embryo rescue technique showcased in this study holds promise for application in other plant species characterized by postzygotic cross-incompatibility.

## Introduction

The genus *Arachis* belongs to the family Leguminosae and is divided into nine sections (Krapovickas *et al.* 2007). It currently consists of 83 described species (Williams 2022). As a member of the Arachis section and the only cultivated species in the genus, *A. hypogaea* L. is valued for oil, food, and feed, and occupies an important place in human and animal nutrition (Badigannavar and Mondal 2023). Other members inside the Arachis section are generally termed as compatible species for ease of success when hybridized with the cultigen. Wild relatives from the remaining eight sections, however, are referred to as incompatible species due to failure in production of hybrid seeds when crossed with *A. hypogaea* L. by conventional means (Wang and Zhang 2013). Cross-incompatibility is manifested by delayed fertilization, low fertilization rates, retarded peg growth, and empty pods with only aborted seed residues. While pre-fertilization barriers do exist, in the genus *Arachis*, the main obstacle is believed to be embryo failure after syngamy.

In their native habitats, wild *Arachis* species can be found in places varying from wetlands or seasonally inundated areas with heavy soils to dry environments with deep sandy or rocky soils (Williams 2022). Adapted to surviving in diverse and harsh natural environments, wild species, especially incompatible species, have evolved high and multiple resistance to biotic and abiotic stresses, including resistance to drought, flooding, salinity, high pH, shading and pests and diseases (Radhakrishnan *et al.* 2022, Wang and Zhang 2013). In addition, some wild species have been identified as high in oil or protein. *A. pintoi* (Caulorrhizae section) and *A. glabrata* (Rhizomatosae section) are commercially utilized as forage crops, while *A. repens* (Caulorrhizae section) is used as ornamental groundcover and a green fertilizer crop (Williams 2022).

If the incompatibility in intersectional crosses can be overcome, it would be of interest not only for the genetic improvement of the cultivated peanut (Cason *et al.* 2023), but perhaps for the breeding of wild peanuts as pastures, groundcovers, landscape ornamentals and forage crops.

*A. glabrata* is widely recognized as a wild *Arachis* species with high and multiple biotic/abiotic stress resistance (Mallikarjuna 2003). Previously, hybrids between Silihong (a Valencia type cultivar) and *A. glabrata* were achieved through *in vitro* peg culture at our laboratory (Shen and Wang 1992, Shen *et al.* 1995). *In vitro* culture of pegs is advantageous over *in vitro* culture of ovules/embryos in that it can save young hybrid embryos that would otherwise abort early in their developmental stages (Shen and Wang 1992). Nevertheless, all these *in vitro* culture techniques are not only time-consuming but also require some skills and equipment, such as the preparation of culture media, aseptic operation techniques, and a growth chamber, and therefore a relatively high monetary input is needed. The *in situ* embryo rescue (ISER) technique developed at our laboratory proved to be simple and effective for recovering hybrids from four incompatible crosses involving three wild species including *A. paraguariensis* Chodat & Hassl. (Wang *et al.* 2020), but has not yet been applied to *A. glabrata*.

The aim of this study was to use the technique, based on previous hormone assays (Li *et al.* 2023), to attempt to obtain intersectional hybrids between peanut cultivars and incompatible wild species including *A. glabrata*, and to provide molecular evidence for hybrid authenticity.

## Materials and Methods

### Plant materials and hand-crossing

Four high-oleic peanut cultivars (Huayu 665, Huayu 668, Huayu 961 and Huayu 965) (Wang *et al.* 2021) and two wild incompatible species (*A. paraguariensis* ssp. *paraguariensis* PI 331187 (2n = 2x = 20) from Section Erectoides and *A. glabrata* (2n = 4x = 40) from Section Rhizomatosae) were used to made five crosses (Table 1). To make the flowering period of the parents meet, male and female parents were planted at Shandong Peanut Research Institute Laixi Experimental Station on May 10 and 26, 2022, respectively. Hand-crossing was carried out using the procedure described previously (Wang *et al.* 2021).

### In situ embryo rescue

**Fig. 1.**
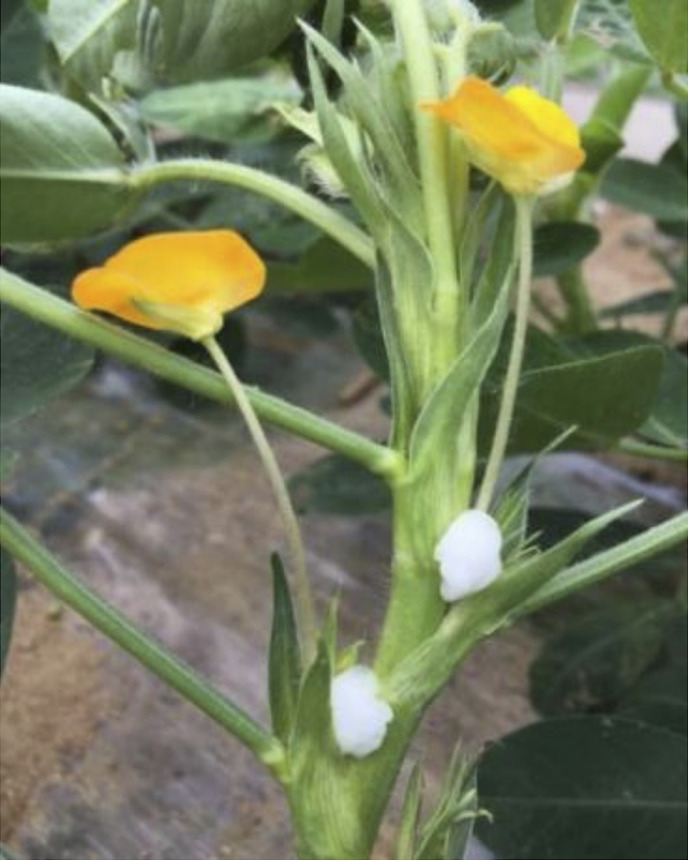
Treatment of pollinated flower bases with hormone-soaked cotton balls.

Following pollination, flower bases were subjected to three different hormone treatments (Tables 1, 2). The hormone treatment solution contained growth hormone, gibberellin and cytokinin (Table 2). In more detail, hormone-soaked cotton balls were applied to the bases of pollinated flowers to rebalance hormones to rescue young hybrid embryos from early abortion, and in this way hybrid seeds might be realized (Fig. 1). No other *in situ* embryo rescue operations were performed other than the initial hormone treatment. The components of the hormone treatment solutions were listed in Table 2. Hybrid seeds (F_1_) were harvested on September 27, 2022.

### NIRS (Near-infrared spectroscopy) screening

As in the hybrid combinations, female parents were high-oleic, and male wild parents were low-oleic, true hybrids would be mid-oleic. Dried individual single peanut seeds were scanned once only using an MPA Fourier transform NIR (Near-infrared) spectrometer (Bruker Optics, Germany). Oleic acid content was predicted with a NIR model developed at our laboratory for the individual single seeds (Han *et al.* 2023). Seeds with oleic acid content predicted by NIRS to be below 72% were retained for further molecular identification.

### Identification of true hybrids by FAD2B (Fatty acid desaturase 2B) KASP (Kompetitive allele specific PCR) assay and transposon element (TE) markers

Genomic DNAs were extracted from peanut cotyledonary slices following the method described by Yu *et al.* (2010). The authenticity of the resultant hybrid seeds was firstly assayed by the KASP technology targeting the F435 type and wild type *FAD2B* (Table 3). KASP reaction mixture (1.6 μl) contained 0.8 μl of DNA template (3.52 ng), 0.4 μl of 2 × Master Mix (LGC, Hoddesdon, UK), and 0.022 μl of primers (Primer FAM (100 μM): Primer HEX (100 μM): Primer common (100 μM): Tris-HCl = 23:6:6:15). KASP PCR profile consisted of denaturation at 94°C for 15 min, 10 cycles of touchdown phase at 94°C for 20 s and at 61°C for 60 s with a 1°C decrease in temperature per cycle, followed by 26 cycles of 94°C for 20 s and 55°C for 60 s. Male and female parents were genotypically pure, and true hybrids had both parental *FAD2B* genotypes (Tables 3, 4). TE primer pairs were used to confirm the hybridity originally identified by KASP assay (Table 5). Procedures for genotyping with TE markers were the same as previously reported (Wang *et al.* 2022).

### Photographing of plants and pods

The F_1_ plants were sown on May 8, 2023. The photographing of F_1_ hybrid plants was carried out at harvest (September 16, 2023). Photos of pods were taken after the pods were dried.

### Statistical analysis

Contingency table chi-square test for comparing the effectiveness of two hormone formulations (A and B) (Table 2) was conducted using the DPS 14.50 package (Tang and Zhang 2013).

## Results

Five intersectional crosses were made using two wild incompatible species and four high-oleic peanut varieties (Table 1). A total of 76 pods and 104 seeds were harvested after hormone treatment of 908 pollinated flower bases (Tables 6, 7).

**Fig. 2.**
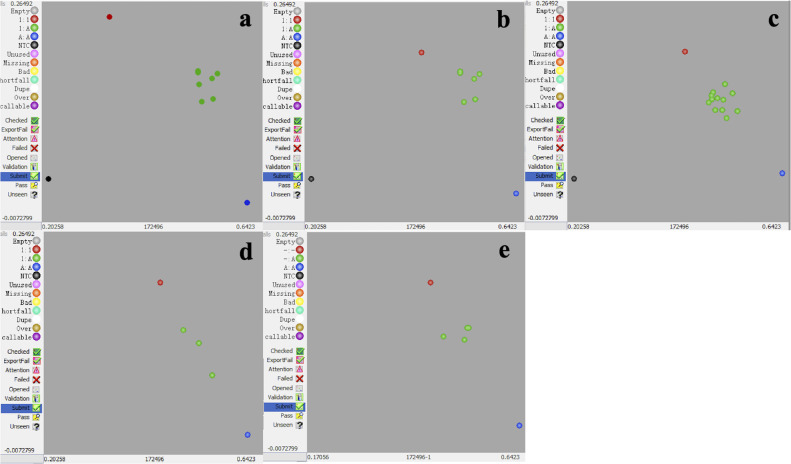
KASP genotyping of *FAD2B* of parents (red and blue dots) and true hybrids (green dots) in 5 cross combinations. a: C2211C (Huayu 961 × *A*. *glabrata*). b: C2213A and C2213B (Huayu 668 × *A. paraguariensis* ssp. *paraguariensis*). c: C2214A and C2214B (Huayu 665 × *A. paraguariensis* ssp. *paraguariensis*). d: C2215A and C2215B (Huayu 961 × *A. paraguariensis* ssp. *paraguariensis*). e: C2216A and C2216B (Huayu 965 × *A. paraguariensis* ssp. *paraguariensis*).

In C2211, C2213, C2214, C2215 and C2216, out of 36, 23, 18, 15 and 12 resultant seeds, 29, 6, 14, 3 and 9 were kept after NIRS screening. However, only 6, 6, 12, 3 and 4 true hybrids were identified by genotyping of *FAD2B* with KASP respectively (Fig. 2) (Tables 6, 7). In Fig. 2b and Fig. 2e, the green dots representing true hybrids overlapped, causing the number of green dots not to match the actual number of true hybrids.

**Fig. 3.**
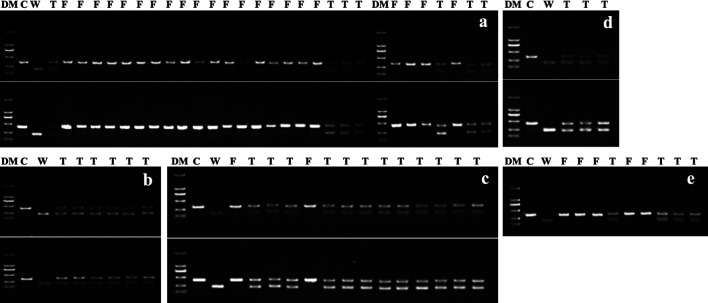
Identification of true hybrids (T) and false hybrids (F) with TE markers in intersectional crosses. DM: D2000 DNA marker (Solarbio, Beijing). C: Cultivar (female parent), W: wild species (male parent). a: C2211, C = Huayu 961, W = *A*. *glabrata*. Top and bottom half of the agarose gel electrophoresis profiles were for primer pairs AhTE0563 and AhTE0552, respectively. b: C2213, C = Huayu 668, W = *A. paraguariensis* ssp. *paraguariensis*. Top and bottom half of the agarose gel electrophoresis profiles were for primer pairs AhTE0437 and AhTE0552, respectively. c: C2214, C = Huayu 665, W = *A. paraguariensis* ssp. *paraguariensis*. Top and bottom half of the agarose gel electrophoresis profiles were for primer pairs AhTE0552 and AhTE0563, respectively. d: C2215, C = Huayu 961, W = *A. paraguariensis* ssp. *paraguariensis*. Top and bottom half of the agarose gel electrophoresis profiles were for primer pairs AhTE0552 and AhTE0563, respectively. e: C2216, C = Huayu 965, W = *A. paraguariensis* ssp. *paraguariensis*. Gel electrophoresis profiles were for AhTE0552.

Twenty-four TE primer pairs were screened to identify usable primers that produced reproducible and discernable male parent-specific bands when male and female parents were tested. Of these, only three pairs of TE primers, AhTE0437, AhTE0552 and AhTE0563, met the requirements. For C2216, only one TE primer pair (AhTE0552) was selected (Table 6, Fig. 3). For the rest of crosses, two TE primer pairs were identified (Table 6, Fig. 3). TE genotyping results were in full agreement with the outcome of KASP assay (Table 6, Figs. 2, 3).

No. of true hybrids/No. of flowers treated averaged 3.41%, ranging from 1.01% (C2215A) to 7.78% (C2214B) (Table 7). No. of true hybrids/No. of resultant seeds averaged 29.81%, varying from 11.67% (C2211C, CC2216A) to 71.43% (C2214A) (Table 7). Thus, among the four high-oleic peanut varieties, Huayu 665 (C2214) appeared to be the best female parent with a high success rate (Table 7). In terms of the ratio of true hybrids to the number of pollinated flowers treated, hormone formulation B was higher than formulation A in three crosses (p < 0.01), and formulation A was higher than formulation B in only one cross (p < 0.01), indicating that in most cases, hormone formulation B was more effective (Table 7). In this study, it was found that tZR outperformed BAP overall.

For the first time, we obtained hybrid seeds of *A. glabrata* with a peanut cultivar by the simple and easy-to-follow *in situ* embryo rescue technique, and the authenticity of the hybrids was consistently supported by the results of KASP and TE marker analyses.

**Fig. 4.**
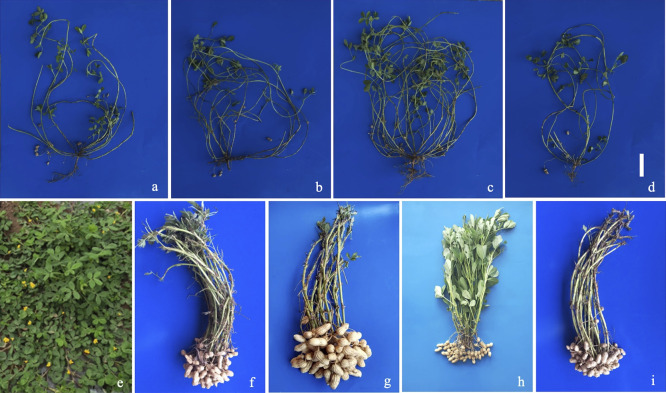
F_1_ hybrid plants (a to d) and their male (e) and female parents (f to i). a: C2211 (Huayu 961 × *A*. *glabrata*), b: C2213 (Huayu 668 × *A. paraguariensis* ssp. *paraguariensis*), c: C2214 (Huayu 665 × *A. paraguariensis* ssp. *paraguariensis*), d: C2216 (Huayu 965 × *A. paraguariensis* ssp. *paraguariensis*). bar length (only for a to d) = 20 cm, e: *A. glabrata*, f: Huayu 961, g: Huayu 668, h: Huayu 665, i: Huayu 965. Note: Unlike the photos of the hybrids, the photos of the parents were not taken from the same distance from the target. Therefore, plant height and pod size of different parents were not comparable in the images.

**Fig. 5.**
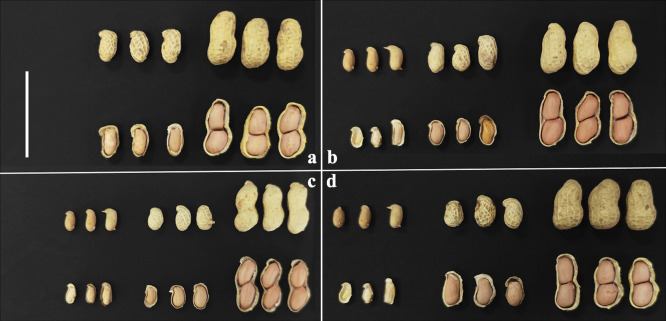
Pods from F_1_ hybrid plants and female and male parents. Female parents (far left), hybrids (second from the left), male parents (third from the left, *A. glabrata* unshown due to no pod set). a: C2211 (Huayu 961 × *A*. *glabrata*), b: C2213 (Huayu 668 × *A. paraguariensis* ssp. *paraguariensis*), c: C2214 (Huayu 665 × *A. paraguariensis* ssp. *paraguariensis*), d: C2216 (Huayu 965 × *A. paraguariensis* ssp. *paraguariensis*). bar length = 5 cm.

The mature F_1_ hybrid plants from four of the five crosses were shown in Fig. 4. C2215 only yielded diseased F_1_ plants, and no photos were taken for them. The female parents Huayu 961, Huayu 668, Huayu 665 and Huayu 965 were erect in growth habit, while the hybrids exhibited prostrate growth habit, and had small pods (Figs. 4, 5). The phenotypes further confirmed the hybridity of the resultant hybrid plants.

## Discussion

In the present study, some seeds were identified as false hybrids despite low NIR oleic acid content predictions, mainly because the NIR model was developed using well filled seeds, and here some seeds were poorly filled, resulting in inaccurate NIR measurements. Nonetheless, NIR screening reduced the workload of molecular identification.

According to our observations on peg culture, the manifestation of incompatibility within the genus *Arachis* was evident not only in the challenges encountered in obtaining hybrid seeds but also in the aberrant development of intersectional hybrid seedlings. Seeds recovered from *in vitro* peg culture exhibited abnormalities, including precocious germination, the hindrance of hybrid seed germination by thick seed coats, necrosis of terminal buds in seedlings, and the darkening of radicles (C.T. Wang, unpublished data). Similarly, scientists from the International Crops Research Institute for the Semi-Arid Tropics (ICRISAT) noted low fertility, mortality of intersectional hybrid F_1_ plants and the inability to produce fully mature BC_1_ seeds, necessitating *in vitro* embryo germination (ICRISAT 1990, Mallikarjuna 2003). It is believed that the success of *in vitro* embryo rescue (IVER) depends on appropriate hormone formulations and early intervention. In ISER, the hormone formulations used are based on our experience with *in vitro* peg culture, and hormones are applied near the ovaries following cross-pollination. While *in vitro* peg culture can be performed 7–10 days after pollination, *in vitro* ovule/embryo rescue requires several weeks to implement, as it can only be done when the ovules or embryos are large enough for dissection and culture. As a result, ISER benefits from earlier intervention and often achieves significantly better outcomes.

Our earlier work in the late 1990s on the use of *A. glabrata* by peg culture produced fertile progenies from only one hybrid seed. The (Silihong × *A. glabrata*) F_1_ hybrid plant grew slowly at its initial developmental stage, resembling its wild species parent (Shen and Wang 1992). Subsequent investigations on the use of wild species via *in situ* embryo rescue unveiled a prevalence of low-frequency sterile plants and trait segregation among F_1_ plants. This segregation likely stems from genomic incompatibility between wild and cultivated species, while the heightened hybrid fertility may be attributed to timely hormonal intervention immediately post-pollination. Notably, certain intersectional F_1_ hybrids closely resembled their cultivar female parents (C.T. Wang and C.J. Jiang, unpublished data), suggesting the potential for breeding interspecific varieties within a relatively brief timeframe. The segregation of traits among F_1_ hybrids underscores the imperative to upscale *in situ* embryo rescue efforts to enhance the possibility of obtaining desirable F_1_ individuals. If traits segregation in F_1_ hybrids is due to chromosome elimination, as suggested by marker losses in our unpublished research, and if it occurs frequently, there is a high likelihood that some KASP and TE assay negative seeds with low oleate content may actually be true hybrids. This highlights the need to refine the molecular identification procedures for peanut intersectional hybrids to avoid missing true hybrids.

Our study achieved intersectional hybrids of *A. glabrata* and *A. paraguariensis* ssp. *paraguariensis* with four high-oleic peanut varieties via *in situ* embryo rescue, confirmed by molecular markers and phenotypic traits. The 31 true intersectional hybrids obtained in this study, including six hybrids between cultivars and *A. glabrata*, facilitate broader observation of hybrid performance encompassing botanical features, agronomic traits, fertility, and cytogenetic characteristics.

Our forthcoming efforts will focus on integrating additional incompatible wild *Arachis* species to broaden their utility. Concurrently, we will undertake thorough cytological and molecular analyses of diverse *Arachis* species genomes. These investigations will inform the strategic incorporation of valuable genes from wild species into cultivated varieties, thereby bolstering crop improvement endeavors.

This *in situ* embryo rescue approach streamlines the utilization of wild, incompatible *Arachis* species rich in resistance and other beneficial traits, essential for broadening the gene pool of peanut breeding. Additionally, it holds promise for the utilization of wild *Arachis* species as groundcover or landscape plants and in pasture applications.

This study highlights the applicability of *in situ* embryo rescue techniques within the genus *Arachis*. Considering the minimal hormone treatments applied in this study, there exists potential to enhance the success rate of *in situ* embryo rescue through additional hormone interventions. Technique may also prove effective for other species facing postzygotic cross-incompatibility barriers, offering significant advantages in distant hybridization breeding endeavors.

## Author Contribution Statement

C.T.W., C.J.J., H.J.S., G.D.Y., M.J.Z. and X.Q.L. designed and coordinated the project. J.K.L. and H.J.S. performed hand-crossing and hormone treatment of pollinated flowers. C.J.J., Z.W.W. and W.J.Q. conducted NIRS analysis, KASP/TE genotyping and photographing. C.J.J. and C.T.W. prepared the manuscript. All authors read and approved the final manuscript.

## Figures and Tables

**Table 1.** Cross combinations and hormone treatments

Cross combination	Hormone treatment no.
C2211: Huayu 961 × *A. glabrata*	C
C2213: Huayu 668 × *A. paraguariensis* ssp. *paraguariensis*	A, B
C2214: Huayu 665 × *A. paraguariensis* ssp. *paraguariensis*	A, B
C2215: Huayu 961 × *A. paraguariensis* ssp. *paraguariensis*	A, B
C2216: Huayu 965 × *A. paraguariensis* ssp. *paraguariensis*	A, B

Note: Huayu 961, Huayu 965, Huayu 665 and Huayu 668 were all high-oleic peanut varieties with F435 type *FAD2B*. Hormone formulations for hormone treatments A, B and C were listed in Table 2.

**Table 2.** Hormone formulations for *in situ* embryo rescue

Treatment no.	Hormone component (mg/L)			
	IAA	GA	BAP	tZR
A	4	4	4.68	0
B	4	4	0	4.68
C	7.36	4	0	3

Note: Hormones and their suppliers. IAA (indole acetic acid): Biotopped, Beijing, GA (gibberellic acid) and BAP (benzylaminopurine): Coolaber, Beijing, and tZR (trans-zeatin riboside): Rhawn, Shanghai.

**Table 3.** KASP primer sequence and *FAD2B* allele genotyping information

Primer name	Sequence (5ʹ-3ʹ)	AlleleX	AlleleY
Primer FAM (*Primer_AlleleX with FAM tail*)	gaaggtgaccaagttcatgctgacaaacacttcgtcgcggtct	A	
Primer HEX (*Primer_AlleleY with HEX tail*)	gaaggtcggagtcaacggattacaaacacttcgtcgcggtcg		–
Primer common	gccgccaccactccaacaca		

Note: Primer FAM and Common Primer amplify *FAD2B* with 441_442 A insertion. Primer HEX and Primer common amplify *FAD2B* without 441_442 A insertion. In AlleleY column, “–” means no 441_442 A insertion in *FAD2B*. *FAD2B* was reported to be located at 154,049,683- to 154,048,544-bp map position on chromosome 19 of the cultivated peanut.

**Table 4.** KASP genotyping of parents in hybrid combinations

Parent	Cultivars/species	KASP genotyping results
Female parent	Huayu 961, Huayu 668, Huayu 665, Huayu 965	A:A
Male parent	*A. glabrata*, *A. paraguariensis* ssp. *paraguariensis*	–:–

Note: In KASP genotyping results column, “–” means no 441_442 A insertion in *FAD2B*.

**Table 5.** Sequences of 24 TE primer pairs used

Primer name	Forward primer sequence (5ʹ-3ʹ)	Reverse primer sequence (5ʹ-3ʹ)
AhTE0426	caacccatgatttgtgaattaag	tgactacaatgtttggtcattttg
**AhTE0437**	**tggcttttgggtgtgtatga**	**gccacgagagaatccaaaaa**
AhTE0443	ttggcctttgatacctgctc	tgaacgcaggaaggaagatt
AhTE0444	ataatgccaccaaagaacgc	tttcacgtacgtactgccca
AhTE0445	acactgctcgcagtttgaga	aaagcaggtgattagtgttacctt
AhTE0457	ttgcgcaatttagcagagatt	ccacctttcattatcccctc
AhTE0478	tgaagcagccacaccatact	gacggttgactaaaaatgttgg
AhTE0481	ttttgtgtgtgctcccgtag	aaatttgttagttagttaggagaaga
AhTE0537	gcatgtttaagcgggtgatt	tgattttcatacgctgttgact
AhTE0540	ccactagactgaaggttggttg	agttcgatggtagtgacccg
AhTE0541	tgttgacaactcactcggga	cattttcaagatgtgttccttga
**AhTE0552**	**gcaacaaaaattctcgaaagc**	**tctgcttatgcttcctcctacc**
AhTE0553	catgcatggaccttaccttg	acaggaggagaagcagcctt
AhTE0555	acaagtcaaattccttcgca	tttgccacttaggcgtcttt
AhTE0556	gatagatggtttgacaagtggg	cataggcctcatcccatgtaa
AhTE0559	ttgctctgacaaccaagctg	gagtcgtttaatcggctattcg
AhTE0544	ttgtttttgttagaagaggcgg	tttttgccattcatactttttgg
AhTE0545	ccactctccggtaacttgga	tcccctcatttaaacatgcc
AhTE0549	tggttaccgaaagatcagaaaaa	aatgcacgtcgacactcaaa
AhTE0561	aatcccaaacaggcaaacac	gagagagtggccattgaaaaa
**AhTE0563**	**tgagaagtaaccccacaagg**	**gaggatttcaagacgatggc**
AhTE0621	cactttggagtttggacagaaa	cgaatcttgatcgcatctctc
AhTE0622	ggtggtgcaaattggaaaaa	gggagtacgtgcgacaattt
AhTE0623	gaagagggggatgatgatga	gacaaacacaacaatctcaagga

Note: Three primer pairs in bold were selected for hybrid identification. Source: Peanut Marker Database (http://marker.kazusa.or.jp/app/marker_list.php?crop=peanut&type=Transposable%20Element).

**Table 6.** Identification of true intersectional hybrids by KASP and TE markers

Cross, hormone treatment and seed serial number	Oleate (%)	KASP genotyping results	KASP hybridity	TE primers	Male parent-specific TE marker	TE hybridity
C2211C-0	51.56	–:A	T	0552, 0563	+	T
C2211C-3	63.55	A:A	F	0552, 0563	–	F
C2211C-9	49.18	A:A	F	0552, 0563	–	F
C2211C-10	69.03	A:A	F	0552, 0563	–	F
C2211C-13	63.14	A:A	F	0552, 0563	–	F
C2211C-14	66.67	A:A	F	0552, 0563	–	F
C2211C-16	58.33	A:A	F	0552, 0563	–	F
C2211C-17	67.97	A:A	F	0552, 0563	–	F
C2211C-18	62.25	A:A	F	0552, 0563	–	F
C2211C-23	61.15	A:A	F	0552, 0563	–	F
C2211C-26	70.44	A:A	F	0552, 0563	–	F
C2211C-27	65.80	A:A	F	0552, 0563	–	F
C2211C-33	67.84	A:A	F	0552, 0563	–	F
C2211C-35	61.19	A:A	F	0552, 0563	–	F
C2211C-36	62.10	A:A	F	0552, 0563	–	F
C2211C-38	60.76	A:A	F	0552, 0563	–	F
C2211C-48	61.56	A:A	F	0552, 0563	–	F
C2211C-49	69.03	A:A	F	0552, 0563	–	F
C2211C-56	54.23	A:A	F	0552, 0563	–	F
C2211C-62	52.05	–:A	T	0552, 0563	+	T
C2211C-63	50.47	–:A	T	0552, 0563	+	T
C2211C-65	45.86	–:A	T	0552, 0563	+	T
C2211C-70	68.76	A:A	F	0552, 0563	–	F
C2211C-80	66.27	A:A	F	0552, 0563	–	F
C2211C-81	69.37	A:A	F	0552, 0563	–	F
C2211C-87	44.04	–:A	T	0552, 0563	+	T
C2211C-94	68.03	A:A	F	0552, 0563	–	F
C2211C-117	46.23	–:A	T	0552, 0563	+	T
C2211C-123	49.27	–:A	T	0552, 0563	+	T
C2213A-1	47.26	–:A	T	0437, 0552	+	T
C2213A-3	50.22	–:A	T	0437, 0552	+	T
C2213A-6	56.09	–:A	T	0437, 0552	+	T
C2213A-7	54.39	–:A	T	0437, 0552	+	T
C2213B-1-2	45.71	–:A	T	0437, 0552	+	T
C2213B-2-2	49.21	–:A	T	0437, 0552	+	T
C2214A-0	49.17	A:A	F	0552, 0563	–	F
C2214A-1	45.28	–:A	T	0552, 0563	+	T
C2214A-2	55.37	–:A	T	0552, 0563	+	T
C2214A-3	56.62	–:A	T	0552, 0563	+	T
C2214A-4	61.15	A:A	F	0552, 0563	–	F
C2214A-5	52.60	–:A	T	0552, 0563	+	T
C2214A-6	48.72	–:A	T	0552, 0563	+	T
C2214B-0	55.34	–:A	T	0552, 0563	+	T
C2214B-1	47.86	–:A	T	0552, 0563	+	T
C2214B-2	54.29	–:A	T	0552, 0563	+	T
C2214B-3	53.84	–:A	T	0552, 0563	+	T
C2214B-4	49.61	–:A	T	0552, 0563	+	T
C2214B-6	52.21	–:A	T	0552, 0563	+	T
C2214B-7	53.17	–:A	T	0552, 0563	+	T
C2215A-4	55.92	–:A	T	0552, 0563	+	T
C2215B-3	54.07	–:A	T	0552, 0563	+	T
C2215B-4	51.95	–:A	T	0552, 0563	+	T
C2216A-0	44.44	A:A	F	0552	–	F
C2216A-1	46.06	A:A	F	0552	–	F
C2216A-2	40.46	A:A	F	0552	–	F
C2216A-3	48.47	–:A	T	0552	+	T
C2216A-5	51.74	A:A	F	0552	–	F
C2216B-1-1	44.32	A:A	F	0552	–	F
C2216B-1-3	48.16	–:A	T	0552	+	T
C2216B-2-0	47.98	–:A	T	0552	+	T
C2216B-2-1	45.96	–:A	T	0552	+	T

Note: In KASP genotyping results column, “–” means no 441_442 A insertion in *FAD2B*. F = false hybrid, T = true hybrid.

**Table 7.** Summary of hormone treated pollinations, pods harvested, resultant seeds and true hybrids

Cross combinations and hormone treatments	Cross	No. of flowers treated	No. of pods harvested	No. of resultant seeds	No. of seeds used for genotyping	No. of true hybrid seeds	No. of true hybrids/ No. of flowers treated (%)	No. of true hybrids/ No. of resultant seeds (%)
C2211C	Huayu 961 × *A. glabrata*	125	23	36	29	6	4.80	16.67
C2213A	Huayu 668 × *A. paraguariensis* ssp. *paraguariensis*	112	9	13	4	4	3.57	30.77
C2213B	Huayu 668 × *A. paraguariensis* ssp. *paraguariensis*	108	8	10	2	2	1.85	20.00
C2214A	Huayu 665 × *A. paraguariensis* ssp. *paraguariensis*	108	6	7	7	5	4.63	71.43
C2214B	Huayu 665 × *A. paraguariensis* ssp. *paraguariensis*	90	11	11	7	7	7.78	63.64
C2215A	Huayu 961 × *A. paraguariensis* ssp. *paraguariensis*	99	4	5	1	1	1.01	20.00
C2215B	Huayu 961 × *A. paraguariensis* ssp. *paraguariensis*	102	7	10	2	2	1.96	20.00
C2216A	Huayu 965 × *A. paraguariensis* ssp. *paraguariensis*	86	4	6	5	1	1.16	16.67
C2216B	Huayu 965 × *A. paraguariensis* ssp. *paraguariensis*	78	4	6	4	3	3.85	50.00
Total		908	76	104	61	31	3.41	29.81

## References

[B1] Badigannavar, A.M. and S. Mondal (2023) Advances in Mutation Breeding of Groundnut (*Arachis hypogaea* L.). *In:* Penna, S. and S.M. Jain (eds.) Mutation Breeding for Sustainable Food Production and Climate Resilience, Springer Nature Singapore, Singapore, pp. 487–519.

[B2] Cason, J.M., C.E. Simpson, M.D. Burow, S. Tallury, H. Pham and S.W. Ravelombola (2023) Use of wild and exotic germplasm for resistance in peanut. J Plant Regist 17: 1–25.

[B3] Han, H., C. Wang, M. Fu, J.J. Yang, Z.W. Wang, X.Z. Wang, X.S. Sun and Z. Yang (2023) Establishment of 11 near infrared analytical models for main fatty acids in individual single peanut kernels. Chinese Journal of Oil Crop Science 45: 407–412 (in Chinese with English summary).

[B4] ICRISAT (1990) ICRISAT Annual Report 1989. International Crops Research Institute for the Semi-Arid Tropics, Patancheru, Andhra Pradesh, India.

[B5] Krapovickas, A., W.C. Gregory, D.E. Williams and C.E. Simpson (2007) Taxonomy of the genus Arachis (Leguminosae). Bonplandia 16 (Suppl.): 1–205.

[B6] Li, J.K., Z.W. Wang, Z. Yang, G.S. Song, X.Z. Wang and C.T. Wang (2023) Post-pollination endogenous phytohormone levels in reproductive organs in two interspecific *Arachis* crosses differing in compatibility. Plant Growth Regul 99: 195–203.

[B7] Mallikarjuna, N. (2003) Wide Hybridization in Important Food Legumes. *In:* Jaiwal, P.K. and R.P. Singh (eds.) Improvement Strategies of Leguminosae Biotechnology. Springer Netherlands, Dordrecht, pp. 155–171.

[B8] Radhakrishnan, T., A.L. Rathnakumar, M.K. Mahatma, S. Chandramohan and S. Patel (2022) Genetic Resources of Groundnut. *In:* Priyadarshan, P.M. and S.M. Jain (eds.) Cash Crops. Springer International Publishing, Cham, pp. 341–406.

[B9] Shen, F.Y. and C.T. Wang (1992) Character identification of intersectional hybrids in peanut. Oil Crops of China 14: 14–17 (in Chinese with English summary).

[B10] Shen, F.Y., C.T. Wang and S.F. Duan (1995) Aseptic culture of gynophores to obtain peanut intersectional hybrids. Euphytica 81: 245–249.

[B11] Tang, Q.Y. and C.X. Zhang (2013) Data Processing System (DPS) software with experimental design, statistical analysis and data mining developed for use in entomological research. Insect Sci 20: 254–260. 23955865 10.1111/j.1744-7917.2012.01519.x

[B12] Wang, C.T. and J.C. Zhang (2013) Peanut Genetic Improvement. Shanghai Sci & Tech Press, Shanghai, China (in Chinese).

[B13] Wang, C.T., X.Z. Wang, Z.W. Wang, Q.M. Yu, Y.Y. Tang, Q. Wu and S.T. Yu (2020) Realizing hybrids between the cultivated peanut (*Arachis hypogaea* L.) and its distantly related wild species using *in situ* embryo rescue technique. Genet Resour Crop Evol 67: 1–8.

[B14] Wang, C.T., S.T. Yu and L.G. Zhu (2021) High oleic acid peanuts in China. Shanghai Sci & Tech Press, Shanghai, China (in Chinese).

[B15] Wang, C.T., G.S. Song, Z.W. Wang, H.J. Li, H.W. Han, X.Y. Chi, X.Z. Wang and X.S. Sun (2022) Assessment of genetic diversity among Chinese high-oleic peanut genotypes using miniature inverted-repeat transposable element markers. Genet Resour Crop Evol 69: 949–958.

[B16] Williams, D.E. (2022) Global strategy for the conservation and use of peanut genetic resources. Global Crop Diversity Trust, Bonn, Germany.

[B17] Yu, S.T., C.T. Wang, S.L. Yu, X.Z. Wang, Y.Y. Tang, D.X. Chen and J.C. Zhang (2010) Simple method to prepare DNA templates from a slice of peanut cotyledonary tissue for polymerase chain reaction. Electron J Biotechnol 13: 9.

